# Identifying Spatial Clusters of Schistosomiasis in Anhui Province of China: A Study from the Perspective of Application

**DOI:** 10.3390/ijerph120911756

**Published:** 2015-09-18

**Authors:** Liqian Sun, Yue Chen, Henry Lynn, Qizhi Wang, Shiqing Zhang, Rui Li, Congcong Xia, Qingwu Jiang, Yi Hu, Fenghua Gao, Zhijie Zhang

**Affiliations:** 1 Department of Epidemiology and Biostatistics, School of Public Health, Fudan University, Shanghai 200032, China; E-Mails: shelloloh@126.com (L.S.); hslynn@shmu.edu.cn (H.L.); mmssddss@gmail.com (R.L.); 14211020071@fudan.edu.cn (C.X.); jiangqw@fudan.edu.cn (Q.J.); 2 Key Laboratory of Public Health Safety, Ministry of Education, Fudan University, Shanghai 200032, China; 3 Laboratory for Spatial Analysis and Modeling, School of Public Health, Fudan University, Shanghai 200032, China; 4 Faculty of Medicine, School of Epidemiology, Pubic Health and Preventive Medicine, University of Ottawa, 451 Smyth Rd., Ottawa ON K1N 6N5, Canada; E-Mail: ychen@uottawa.ca; 5 Anhui Institute of Parasitic Diseases, Wuhu 230061, China; E-Mails: 14111020001@fudan.edu.cn (Q.W.); 09301020024@fudan.edu.cn (S.Z.)

**Keywords:** schistosomiasis, spatial pattern, spatial clustering, spatial-temporal clustering, China

## Abstract

With the strategy shifting from morbidity control to transmission interruption, the burden of schistosomiasis in China has been declining over the past decade. However, further controls of the epidemic in the lake and marshland regions remain a challenge. Prevalence data at county level were obtained from the provincial surveillance system in Anhui during 1997–2010. Spatial autocorrelation analysis and spatial scan statistics were combined to assess the spatial pattern of schistosomiasis. The spatial-temporal cluster analysis based on retrospective space-time scan statistics was further used to detect risk clusters. The Global Moran’s *I* coefficients were mostly statistically significant during 1997–2004 but not significant during 2005–2010. The clusters detected by two spatial cluster methods occurred in Nanling, Tongling, Qingyang and Wuhu during 1997–2004, and Guichi and Wuhu from 2005 to 2010, respectively. Spatial-temporal cluster analysis revealed 2 main clusters, namely Nanling (1999–2002) and Guichi (2005–2008). The clustering regions were significantly narrowed while the spatial extent became scattered during the study period. The high-risk areas shifted from the low reaches of the Yangtze River to the upper stream, suggesting the focus of schistosomiasis control should be shifted accordingly and priority should be given to the snail habitats within the high-risk areas of schistosomiasis.

## 1. Introduction

Schistosomiasis is a waterborne parasitic disease, which is prevalent in tropical and subtropical areas of the world [1] and 80% of the victims are from sub-Saharan Africa [2]. Among the major parasitic diseases, schistosomiasis is the second most serious one from the perspective of public health, only behind malaria [3]. By 2012, at least 240 million people were infected and almost 700 million people living in the endemic areas were at risk [4]. According to the Global Burden of Disease Study 2010 (GBD 2010), the global burden of schistosomiasis was estimated at 3.31 (95% confidence interval: 1.70–6.26) million disability-adjusted life years (DALYs) [5]. Therefore, the situation of schistosomiasis remains serious.

In China, the critical schistosome species responsible for humans and cattle infections is *Schistosoma japonicum* [6] and its history is more than 2000 years [7,8]. The endemic areas of schistosomiasis are categorized into three types [9,10,11]: (1) the lake and marshland regions; (2) plain regions with waterway networks; and (3) hilly and mountainous regions, where 82% of human cases were reported from the type (1) areas [12]. The Chinese government has been paying a high amount of attention to the control of schistosomiasis, and a multitude of control programs have been implemented over the past 60 years [8,13,14]. The latest two control programs at the national level were the World Bank Loan Project (WBLP) that started from 1992 to 2001 [15], which mainly emphasized praziquantel-based morbidity control on humans and domestic animals [16], and the integrated control program implemented since 2005, which emphasized infection source control [17]. The disease burden, therefore, has been decreased significantly, and the disease transmission has been blocked in many areas that were previously endemic [8,16,18]. However, in some settings, the control of *S. japonicum* remains a challenge, *i.e.*, along the middle and lower reaches of the Yangtze River, where Anhui Province is a typical case [8,18,19,20,21]. Water level is the key influencing factor [22] causing *S. japonicum* to resurge in some areas, even in previously controlled or eliminated areas [19,23,24,25]. Hence, monitoring the spatial changes of schistosomiasis risk is very important for understanding the current situation and making the right control strategies.

With a rapid development of the geographical information systems (GIS), remote sensing (RS) and spatial statistics, an increasing number of public health problems have been analyzed using these methods [26,27,28,29]. Presenting the spatial distribution of a disease with a time sequence from a spatial perspective makes it easy to understand the risk variations of the disease while the spatial-temporal analysis of disease risk is helpful for predicting future epidemics [30]. In this study, statistics of cluster detection were employed to identify the clustering of schistosomiasis in Anhui Province. Firstly, the spatial risk of schistosomiasis was analyzed using the Moran’s *I* spatial autocorrelation statistics and SatScan statistics. Secondly, a spatial-temporal analysis was conducted to identify the risk distribution and to understand the risk variation of schistosomiasis over space and time. Finally, the potential implications of the findings were discussed.

## 2. Experimental Section

### 2.1. Study Area

**Figure 1:**
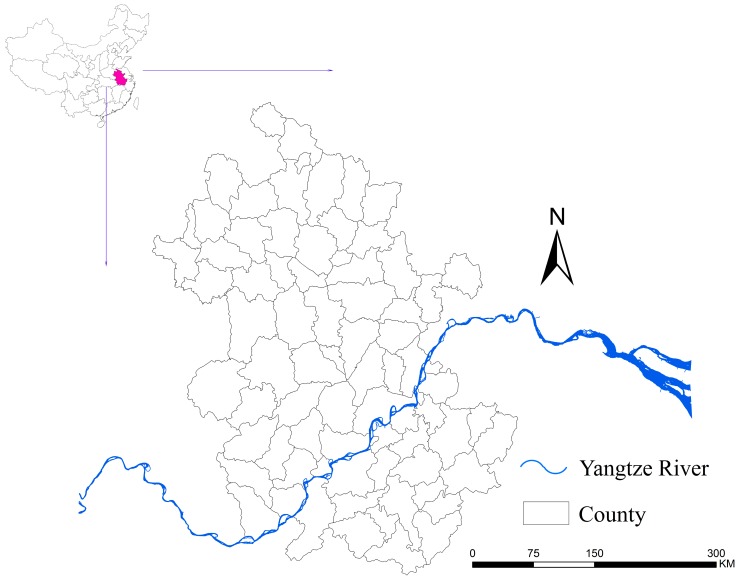
Location of Anhui Province, China. The maps were created using the ArcGIS 10.0 software (ESRI Inc., Redlands, CA, USA).

Anhui Province covers an area of approximately 139,000 km^2^ with a population of 60.83 million (2014), flowing through the lower reaches of the Yangtze River (Figure 1). The climate of southern Anhui Province is humid subtropical monsoon, which is ideal for the survival of *O. hupensis*. Hence, it was, historically, one of the most serious endemic areas of *S. japonicum* in China [31].

### 2.2. Schistosomiasis Prevalence Data

The county-level epidemiological data on schistosomiasis from 1997 to 2010 were provided by the Anhui Institute of Schistosomiasis Prevention and Control. Annual surveys on schistosomiasis were carried out in each village during the study period with the data reported to the townships and finally aggregated at the county level. Individuals aged 5 to 65 years were invited to participate in the survey. All participants were screened for *S. japonicum*-specific immunoglobulin G by using an Indirect Hemagglutination Assay (IHA) test [32]. Seropositive individuals were further tested by the Kato-Katz technique [33]. Residents that were positive for both a serological test and a fecal parasitological test were defined as being infected.

### 2.3. Ethics Statement

The study protocol was approved by the Ethics Committee of Fudan University (ID: IRB#2011-03-0295). Written informed consent was also obtained from all participants.

### 2.4. Statistical Analysis

Firstly, a descriptive statistical analysis was carried out to provide some basic statistics in regard to the prevalence of schistosomiasis using SPSS 17.0 software (SPSS Inc., Chicago, IL, USA).

Secondly, a spatial autocorrelation analysis was executed using ArcGIS 10.0 software (ESRI Inc., Redlands, CA, USA), to determine whether the pattern of schistosomiasis was clustered, dispersed, or random [34]. Global Moran’s *I* statistics were used to test whether the global spatial autocorrelation of schistosomiasis cases existed in each year while local Moran’s *I* statistics [35] were used to detect the location of the clusters of schistosomiasis. The way to measure the spatial relationships (*i.e.*, spatial weight matrix) for both global and local Moran’s *I* is that, if two counties share a common border, the weight element equals 1 and 0, otherwise.

Thirdly, Kulldorff’s scan statistics [36] were employed to detect not only the spatial but also the space-time clustering of schistosomiasis using the SatScan 8.0 software (Kulldorff and Information Management Services, Inc., Boston, MA, USA). To detect the high-risk spatial clusters of cases, the statistics use a moving circular window scanning the study area, and the maximum size of which is no more than 50% of the total population. The relative risk (RR) was determined through comparing observed and expected case numbers inside and outside the scan window to detect clusters [27]. The *p*-value for each cluster is obtained by Monte Carlo hypothesis testing. Compared to the spatial scan statistics, the spatial-temporal scan statistics uses a cylinder-shaped scanning window. The bottom of the cylinder corresponds with spatial units, and the height corresponds with a certain time range. The scanning radius of the window both in the population at risk and study periods was set to 30% in this study. The analysis was carried out using the Poisson probability model, and 999 Monte Carlo replications were used to test the significance.

## 3. Results

**Figure 2:**
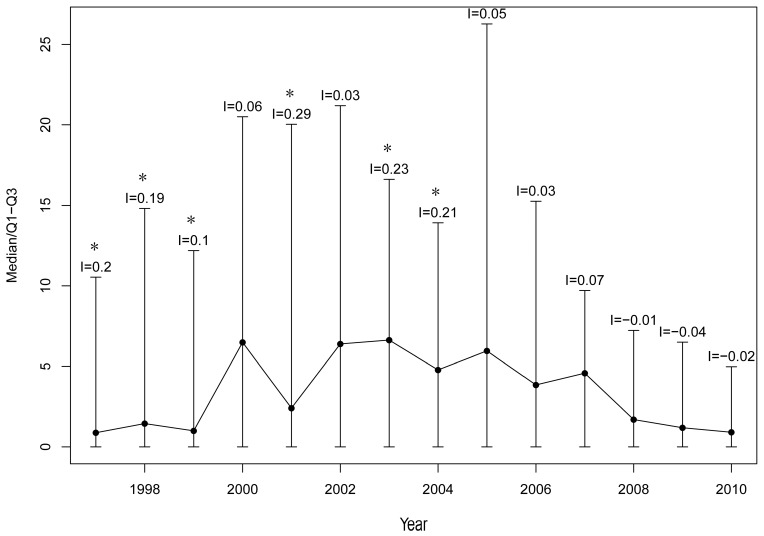
The prevalence (cases/10,000) trend of schistosomiasis and its global spatial autocorrelation, Anhui Province during 1997–2010. The black spot represented the median prevalence of schistosomiasis; the vertical line represented the interquartile range (IQR); *I:* the Global Moran’s *I*; *: *p* < 0.05 (*p-*value for the Global Moran’s *I*).

**Figure 3:**
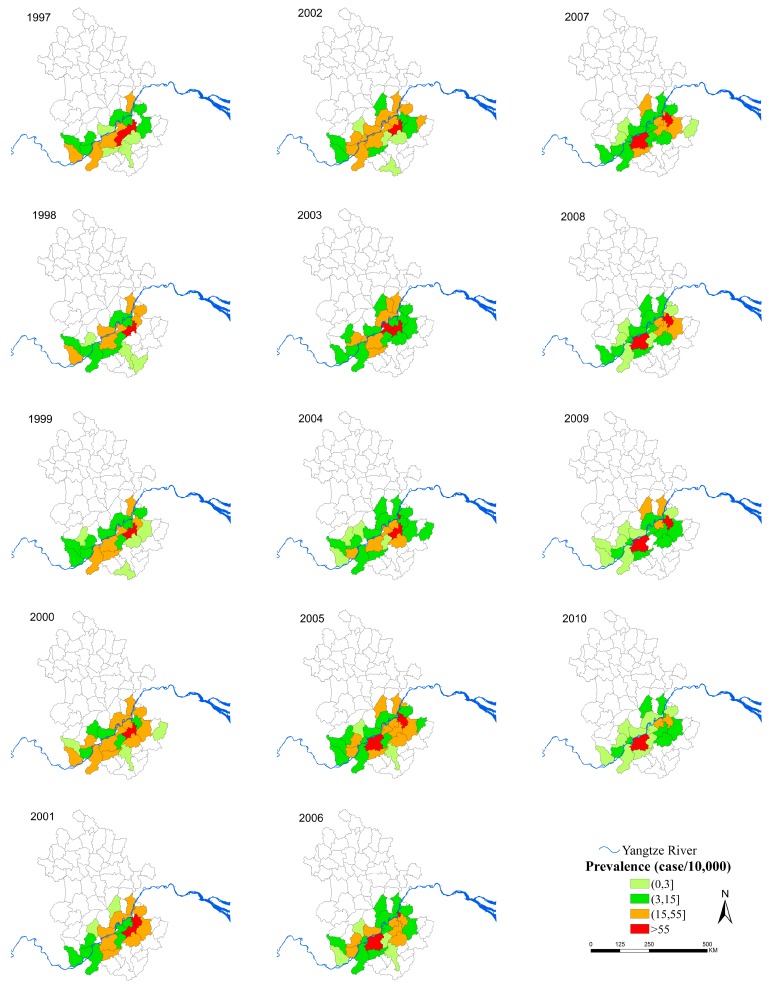
Annual prevalence of schistosomiasis at county level during 1997–2010. The maps were created using the ArcGIS 10.0 software (ESRI Inc., Redlands, CA, USA).

The median prevalence increased from 0.88/10,000 in 1997 to 6.63/10,000 in 2003, and then decreased to 0.91/10,000 in 2010 (Figure 2). Figure 3 shows that there were more areas with a high prevalence (>15/10,000) in 1997–2004 than in 2005–2010. Nanling County (1997–2004), Guichi County and Wuhu County (2005–2010) had a prevalence of >55/10,000, and these counties were along the middle and lower reaches of the Yangtze River. The annual global Moran’s *I* was statistically significant only before 2004.

**Figure 4:**
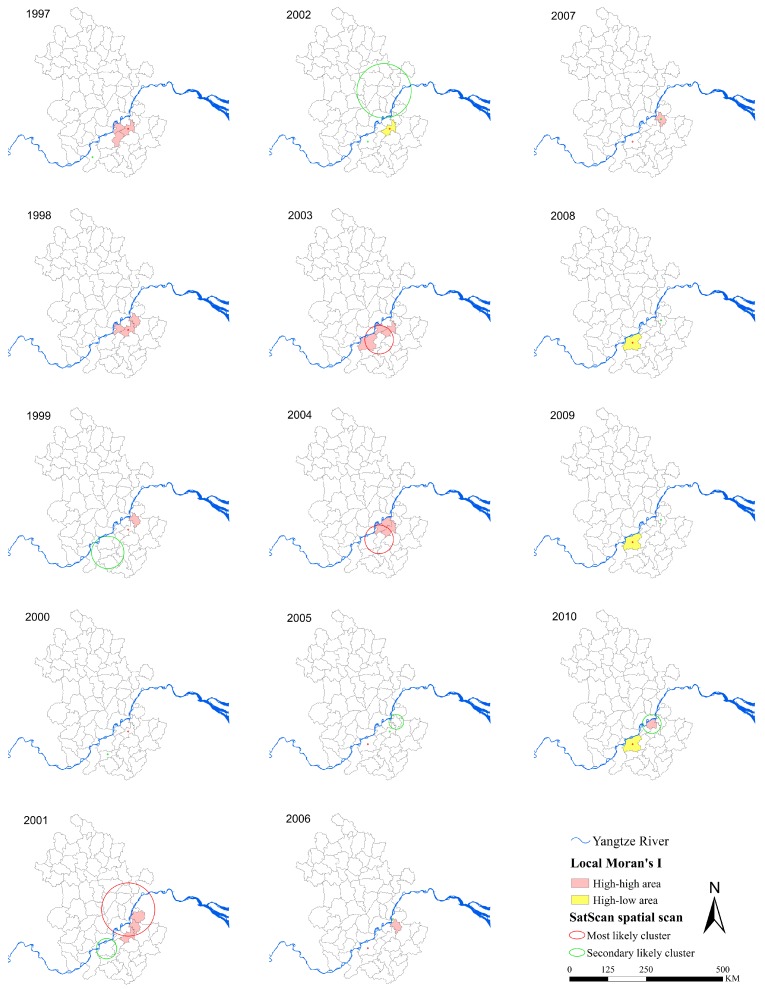
Annual spatial clusters of schistosomiasis during 1997–2010. Each panel shows the results of both methods for each year, the Anselin’s local Moran’s *I* test and the Kulldorff’s spatial scan statistics. The maps were created using the ArcGIS 10.0 software (ESRI Inc., Redlands, CA, USA).

As shown in Figure 4, spatial clusters were detected by both the local Moran’s *I* test and the Kulldorff’s spatial scan statistics. During 1997–2004, the local Moran’s *I* identified seven high-high counties, with an annual number ranging from 0 (in 2000) to 3 (in 1997–1998, 2001 and 2003–2004) and one high-low county (in 2002), and the median annual prevalence of the high-high counties was 72 (IQR = 42–96) cases per 10,000. The spatial scan method identified 14 significant spatial clusters during 1997–2004 (eight most likely clusters and six secondary likely clusters), with an annual number ranging from 1 (in 1998, and 2003–2004) to 3 (in 2002). The median number of counties included in each cluster was one (IQR = 1–5). During 2005–2010, the local Moran’s *I* identified two high-high counties, with an annual number ranging from 0 (in 2005) to 1 (in 2006–2007 and 2010) and one high-low county (in 2008–2010), and the median annual prevalence within these counties was 96 (IQR = 37.5–115.25) cases per 10,000. The Kuldorff’s spatial scan statistics identified 13 significant spatial clusters altogether (six most likely clusters and seven secondary likely clusters), with an annual number ranging from two (in 2006–2010) to three (in 2005); the median number of counties contained within a cluster was one (IQR = 1–1). Over the whole study period, almost 70% of the high-risk counties identified with the local Moran’s *I* were included in clusters detected by SaTScan and 29% of the SaTScan clusters encompassed high-high counties and high-low counties.

## 4. Discussion

Anhui Province was one of the most serious schistosomiasis-endemic provinces in the five lake and marshland regions [37]. In this study, we have investigated the spatial clusters of schistosomiasis in the endemic region of Anhui Province from 1997 to 2010 to explore its spatial dynamic change based on the county-level prevalence data.

In our study, we found that the overall prevalence of schistosomiasis maintained at a low level, but with fluctuations. As shown in Figure 2, the median prevalence as well as IQR increased during 1997–2000, which may relate to the flood in 1998 [38]. After the flood, infected individuals would have an incubation period to become infectious, which led to the delayed effect of flooding [39]. Schistosomiasis occurrence became more apparent three years after flooding. In 2001, the median prevalence clearly was reduced at the end of the WBLP. However, from 2001 to 2003, the data showed that schistosomiasis rebounded shortly after the termination of the WBLP, which also was found in other studies [25,40]. There were more areas inhabited by infected *O. hupensis* and acute human schistosomiasis cases were detected again in some areas [41], suggesting that the WBLP strategy might not have a long-term sustainable effect on controlling schistosomiasis and the possible reasons were the current low endemic levels of infection and the decline of drug compliance rate [18,42,43]. During 2004–2010, the prevalence showed a declining trend, which might have resulted from an integrated control strategy. These findings were consistent with previous studies conducted in Jiangsu and Hubei provinces [17,41].

**Figure 5:**
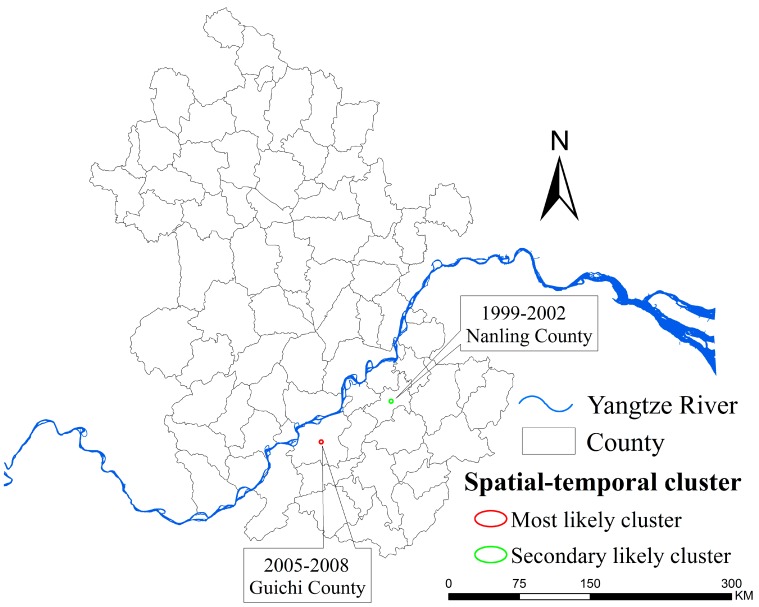
Location of spatial-temporal clustering. The maps were created using the ArcGIS 10.0 software (ESRI Inc., Redlands, CA, USA).

The global Moran’s *I* coefficients were statistically significant during 1997–2004 but not significant during 2005–2010, suggesting that the spatial distribution of the disease changed from a clustered pattern to a scattered one. The implementation of the integrated control strategy might reduce the burden of disease. Furthermore, some years (1998 and 2008, 2000 and 2003) had a similar prevalence but very different spatial distribution, indicating the importance of studying schistosomiasis risk from a spatial perspective.

We used both local Moran’s *I* statistics and Kulldorff’s scan statistics in the current study. Local Moran’s *I* statistics is a method for measuring the spatial autocorrelation of spatial unit and its neighbors. The choice of spatial weight describing the spatial relation of neighbors is subjective, which might lead to lack of certain stability and reliability [44]. Kulldorff’s spatial scan statistics, on the other hand, is a method comparing observed and expected case numbers inside and outside a scanning window. The shape of the moving window needs to be pre-specified while the spatial unit tends to be irregular and complicated in reality. Consistent findings using more than one spatial method suggested robust results [45].

The risk of schistosomiasis, was dramatically reduced from 1997–2004 to 2005–2010. We detected disease clusters in Nanling, Tongling, Qingyang and Wuhu during 1997–2004, and Guichi and Wuhu from 2005 to 2010, respectively. The number of counties within clusters decreased from four to two, but the spatial extent became scattered (as shown in Figure 4) during the two parts of the study period. This pattern might indicate that the integrated control strategy was effective to reduce the risk of schistosomiasis since 2005, conforming to other studies [41,46]. It is well known that the transmission of *S. japonicum* is strongly associated with the distribution of *O. hupensis*—the intermediate host of schistosome [47]. The scattered clusters of schistosomiasis may reflect the dispersed distribution of snail habitats to some extent [48]. Several factors have been suggested as underlying causes of this scattered pattern on snail habitats, including development of roads or highways [49] and ecosystem changes (such as conceding the land to forestry [48], and regular flooding [50]).

Implementation of an effective and sustainable strategy in endemic areas is of great importance in the control of schistosomiasis. Chemotherapy with praziquantel is still one of the main strategies in China’s National Schistosomiasis Control Program since 1980s [51], but the use of praziquantel for humans and domestic animals has been only temporarily effective [43]. Once the chemotherapeutic measure stopped or even just its coverage rate decreased, the prevalence of schistosomiasis would grow again [18]. Hence, the chemotherapy-based control strategy was no longer an ideal method for current low density of infected snails and reduced financial resources [9].

The integrated control strategy mainly focuses on interrupting the snail infection of eggs from cattle or human feces to block the life cycle of schistosomiasis, but the difficulty is that more than 40 mammalian animals could serve as its definite hosts [51]. Since the amphibious snail is the sole intermediate host of *S. japonicum*, controlling snails is central to the control of schistosomiasis [52]. The strategy of snail control with environmental modification and mollusciciding is more effective in low-prevalence areas, for which it is important to identity the hotspots of transmission sites for precise targeted control.

In addition, our spatial-temporal cluster analysis revealed two main clusters, namely Nanling (1999–2002) and Guichi (2005–2008). The spatial clustering of the disease shifted from the low reaches of the Yangtze River to the upper stream. The areas within the upper stream of Yangtze River in Anhui should be targeted for effective the control of schistosomiasis.

## 5. Conclusions

The prevalence of schistosomiasis was dramatically reduced over the study period and remained at a low level in recent years. Two main clusters were found, namely Nanling County (1999–2002) and Guichi County (2005–2008), with a tendency of shifting from the low reaches of the Yangtze River to the upper stream. The overall clustering regions had a tendency of narrowing, but the spatial extent tended to be scattered, suggesting that the snail habitats within clustering regions of schistosomiasis must be targeted for further intervention.

## References

[1] Odhiambo G.O., Musuva R.M., Atuncha V.O., Mutete E.T., Odiere M.R., Onyango R.O., Alaii J.A., Mwinzi P.N. (2014). Low levels of awareness despite high prevalence of schistosomiasis among communities in Nyalenda informal settlement, Kisumu city, western Kenya. PLoS Negl. Trop. Dis..

[2] Ladu R. (2014). Schistosomiasis as a rare cause of recurrent acute appendicitis—A case report. Int. J. Surg. Case Rep..

[3] Xu B., Gong P., Seto E., Liang S., Yang C.H., Wen S., Qiu D.C., Gu X.G., Spear R. (2006). A spatial-temporal model for assessing the effects of intervillage connectivity in schistosomiasis transmission. Ann. Assoc. Am. Geogr..

[4] Shan C., Zhou X., Zhu H. (2014). The dynamics of growing islets and transmission of schistosomiasis Japonica in the Yangtze River. Bull. Math. Biol..

[5] Krishnamurthi R.V., Feigin V.L., Forouzanfar M.H., Mensah G.A., Connor M., Bennett D.A., Moran A.E., Sacco R.L., Anderson L.M., Truelsen T. (2013). Global and regional burden of first-ever ischaemic and haemorrhagic stroke during 1990–2010: Findings from the Global Burden of Disease Study 2010. Lancet Global Health.

[6] Gryseels B., Polman K., Clerinx J., Kestens L. (2006). Human schistosomiasis. Lancet.

[7] Ross A.G., Sleigh A.C., Li Y., Davis G.M., Williams G.M., Jiang Z., Feng Z., McManus D.P. (2001). Schistosomiasis in the People’s Republic of China: Prospects and challenges for the 21st century. Clin. Microbiol. Rev..

[8] Zhou X.N., Wang L.Y., Chen M.G., Wu X.H., Jiang Q.W., Chen X.Y., Zheng J., Utzinger J. (2005). The public health significance and control of schistosomiasis in China—Then and now. Acta Trop..

[9] Zhang Z., Carpenter T.E., Chen Y., Clark A.B., Lynn H.S., Peng W., Zhou Y., Zhao G., Jiang Q. (2008). Identifying high-risk regions for schistosomiasis in Guichi, China: A spatial analysis. Acta Trop..

[10] Yang K., Zhou X.N., Wu X.H., Steinmann P., Wang X.H., Yang G.J., Utzinger J., Li H.J. (2009). Landscape pattern analysis and Bayesian modeling for predicting Oncomelania hupensis distribution in Eryuan County, People’s Republic of China. Amer. J. Trop. Med. Hyg..

[11] Zhou X.N., Bergquist R., Leonardo L., Yang G.J., Yang K., Sudomo M., Olveda R. (2010). Schistosomiasis japonica control and research needs. Adv. Parasitol..

[12] Liu J., Yu H., Shi Y., Li H., He L., Li J., Dong C., Xie Q., Jin Y., Lu K., Lin J. (2013). Seasonal dynamics of schistosoma japonicum infection in buffaloes in the Poyang Lake region and suggestions on local treatment schemes. Vet. Parasitol..

[13] Mao S.B. (1986). Recent progress in the control of schistosomiasis in China. Chin. Med. J. Engl..

[14] Chitsulo L., Engels D., Montresor A., Savioli L. (2000). The global status of schistosomiasis and its control. Acta Trop..

[15] Balen J., Liu Z., McManus D.P., Raso G., Utzinger J., Xiao S., Yu D., Zhao Z., Li Y. (2013). Health access livelihood framework reveals potential barriers in the control of schistosomiasis in the Dongting lake area of Hunan province, China. PLoS Negl. Trop. Dis..

[16] Xianyi C., Liying W., Jiming C., Xiaonong Z., Jiang Z., Jiagang G., Xiaohua W., Engels D., Minggang C. (2005). Schistosomiasis control in China: The impact of a 10-year World Bank Loan Project (1992–2001). Bull. WHO.

[17] Chen Y.Y., Liu J.B., Huang X.B., Cai S.X., Su Z.M., Zhong R., Zou L., Miao X.P. (2014). New integrated strategy emphasizing infection source control to curb schistosomiasis japonica in a marshland area of Hubei Province, China: Findings from an eight-year longitudinal survey. PLoS ONE.

[18] Yuan H., Jiang Q., Zhao G., He N. (2002). Achievements of schistosomiasis control in China. Mem. Inst. Oswaldo Cruz.

[19] Utzinger J., Zhou X.N., Chen M.G., Bergquist R. (2005). Conquering schistosomiasis in China: The long march. Acta Trop..

[20] Zhao G.M., Zhao Q., Jiang Q.W., Chen X.Y., Wang L.Y., Yuan H.C. (2005). Surveillance for schistosomiasis japonica in China from 2000 to 2003. Acta Trop..

[21] McManus D.P., Gray D.J., Li Y., Feng Z., Williams G.M., Stewart D., Rey-Ladino J., Ross A.G. (2010). Schistosomiasis in the People’s Republic of China: The era of the Three Gorges Dam. Clin. Microbiol. Rev..

[22] Basch P.F. (1986). Schistosomiasis in China: An update. Amer. J. Chin. Med..

[23] Lu D.B., Wang T.P., Rudge J.W., Donnelly C.A., Fang G.R., Webster J.P. (2010). Contrasting reservoirs for Schistosoma japonicum between marshland and hilly regions in Anhui, China—A two-year longitudinal parasitological survey. Parasitology.

[24] Liang S., Seto E., Remais J.V., Zhong B., Yang C.H., Hubbard A., Davis G.M., Gu X.G., Qiu D.C., Spear R.C. (2007). Environmental effects on parasitic disease transmission exemplified by schistosomiasis in western China. Proc. Natl. Acad. Sci. USA.

[25] Liang S., Yang C., Zhong B., Qiu D. (2006). Re-emerging schistosomiasis in hilly and mountainous areas of Sichuan, China. Bull. WHO.

[26] Rahman M.R., Shi Z.H., Chongfa C. (2014). Assessing regional environmental quality by integrated use of remote sensing, GIS, and spatial multi-criteria evaluation for prioritization of environmental restoration. Environ. Monit. Assess..

[27] Gomes E.C., Leal-Neto O.B., de Oliveira F.J., Campos J.V., Souza-Santos R., Barbosa C.S. (2014). Risk analysis for occurrences of schistosomiasis in the coastal area of Porto de Galinhas, Pernambuco, Brazil. BMC Infect. Dis..

[28] Martin V., Pfeiffer D.U., Zhou X., Xiao X., Prosser D.J., Guo F., Gilbert M. (2011). Spatial distribution and risk factors of highly Pathogenic Avian Influenza (HPAI) H5N1 in China. PLoS Pathog..

[29] Feo G.D., Gisi S.D. (2014). Using MCDA and GIS for hazardous waste landfill siting considering land scarcity for waste disposal. Waste Manag..

[30] Paireau J., Girond F., Collard J., Mainassara H.B., Jusot J. (2012). Analysing spatio-temporal clustering of meningococcal meningitis outbreaks in Niger reveals opportunities for improved disease control. PLoS. Negl. Trop. Dis..

[31] Liu R., Dong H., Jiang M. (2013). The new national integrated strategy emphasizing infection sources control for schistosomiasis control in China has made remarkable achievements. Parasitol. Res..

[32] Hu Y., Zhang Z., Chen Y., Wang Z., Gao J., Tao B., Jiang Q., Jiang Q. (2013). Spatial pattern of schistosomiasis in Xingzi, Jiangxi province, China: The effects of environmental factors. Parasites Vectors.

[33] Katz N., Chaves A., Pellegrino J. (1972). A simple device for quantitative stool thick-smear tecnique in schistosomiasis mansoni. Rev. Inst. Med. Trop. Sao Paulo.

[34] Yang K., Li W., Sun L., Huang Y., Zhang J., Wu F., Hang D., Steinmann P., Liang Y. (2013). Spatio-temporal analysis to identify determinants of Oncomelania hupensis infection with Schistosoma japonicum in Jiangsu province, China. Parasites Vectors.

[35] Anselin L. (1995). Local indicators of spatial association—LISA. Geogr. Anal..

[36] Kulldorff M. (1997). A spatial scan statistic. Commun. Stat. Theory Methods.

[37] Wang T.P., Zhao F., Zhang S.Q., Zhang Z.J., Gao F.H., Zhou Y.B., He J.X., Jiang Q.W. (2011). Spatial-temporal clustering analysis of schistosomiasis in Anhui from 2000 to 2008. J. Trop. Dis. Parasitol..

[38] Zhou X.N., Dandan L., Huiming Y., Honggen C., Leping S., Guojing Y., Qingbiao H., Brown L., Malone J.B. (2002). Use of landsat TM satellite surveillance data to measure the impact of the 1998 flood on snail intermediate host dispersal in the lower Yangtze River Basin. Acta Trop..

[39] Qi L., Cui J., Huang T., Ye F., Jiang L. (2014). Mathematical model of schistosomiasis under flood in Anhui province. Abstr. Appl. Anal..

[40] Engels D., Wang L., Palmer K. (2005). Control of schistosomiasis in China. Acta Trop..

[41] Sun L.P., Wang W., Liang Y.S., Tian Z.X., Hong Q.B., Yang K., Yang G.J., Dai J.R., Gao Y. (2011). Effect of an integrated control strategy for schistosomiasis japonica in the lower reaches of the Yangtze River, China: An evaluation from 2005 to 2008. Parasites Vectors.

[42] Wang L.D., Guo J.G., Wu X.H., Chen H.G., Wang T.P., Zhu S.P., Zhang Z.H., Steinmann P., Yang G.J., Wang S.P. (2009). China’s new strategy to block schistosoma japonicum transmission: Experiences and impact beyond schistosomiasis. Trop. Med. Int. Health.

[43] Wang L.D., Chen H.G., Guo J.G., Zeng X.J., Hong X.L., Xiong J.J., Wu X.H., Wang X.H., Wang L.Y., Xia G. (2009). A strategy to control transmission of schistosoma japonicum in China. N. Engl. J. Med..

[44] Wang P.A., Luo W.H., Bai Y.P. (2012). Comparative analysis of aggregation detection based on spatial autocorrelation and spatial-temporal scan statistics. Hum. Geogr..

[45] Rainey J.J., Omenah D., Sumba P.O., Moormann A.M., Rochford R., Wilson M.L. (2007). Spatial clustering of endemic Burkitt’s lymphoma in high-risk regions of Kenya. Int. J. Cancer.

[46] Hong X.C., Xu X.J., Chen X., Li Y.S., Yu C.H., Yuan Y., Chen Y.Y., Li R.D., Qiu J., Liu Z.C. (2013). Assessing the effect of an integrated control strategy for schistosomiasis japonica emphasizing bovines in a marshland area of Hubei Province, China: A cluster randomized trial. PLoS Negl. Trop. Dis..

[47] Ofulla A.V., Adoka S.O., Anyona D.N., Abuom P.O., Karanja D., Vulule J.M., Okurut T., Matano A., Dida G.O., Jembe T. (2013). Spatial distribution and habitat characterization of schistosomiasis host snails in lake and land habitats of western Kenya. Lakes Reserv. Res. Manag..

[48] Hu Y., Xiong C.L., Zhang Z.J., Luo C., Cohen T., Gao J., Zhang L.J., Jiang Q.W. (2014). Changing patterns of spatial clustering of schistosomiasis in Southwest China between 1999–2001 and 2007–2008: Assessing progress toward eradication after the World Bank Loan Project. Int. J. Environ. Res. Public Health.

[49] Jia Y.H., Dai D.H., Liu Y. (2005). Performance Analyse and Evaluation of Freeway in China. J. Beijing Jiaotong Univ..

[50] Huang Y.X., Hong Q.B., Gao Y., Gao Y., Zhang L.H., Chen H., Guo J.H., Liang Y.S. (2006). Impact of floodwater on schistosomiasis transmission in area of lower reaches of Yangtze River. Chin. J. Schistosomiasis Control.

[51] Xu J., Xu J., Li S., Zhang L., Wang Q., Zhu H., Zhou X. (2015). Integrated control programmes for schistosomiasis and other helminth infections in P.R. China. Acta Trop..

[52] Lin T., Jiang Q.W., Lin D.D., Chen H.G., Zhao G.M., Liu J.X., Zhang S.J. (2001). Classification study on the marshland in endemic areas of Schistosoma japonicum using satellite TM image data. Chin. J. Prev. Med..

